# Sex Differences in Outcomes of Patients with an Implantable Cardioverter-Defibrillator for the Secondary Prevention of Sudden Cardiac Death

**DOI:** 10.3390/jcdd11040116

**Published:** 2024-04-05

**Authors:** Alwin B. P. Noordman, Michiel Rienstra, Yuri Blaauw, Bart A. Mulder, Alexander H. Maass

**Affiliations:** Department of Cardiology, Heart Center, University Medical Center Groningen, University of Groningen, 9713 GZ Groningen, The Netherlands; a.b.p.noordman@umcg.nl (A.B.P.N.);

**Keywords:** implantable cardioverter-defibrillator, secondary prevention, sex differences, appropriate device therapy, ventricular arrhythmias

## Abstract

**Background:** In patients with an implantable cardioverter-defibrillator (ICD) for secondary prevention, sex differences may exist in clinical outcomes. We sought to investigate sex differences in appropriate ICD therapy, appropriate and inappropriate shock, and all-cause mortality in this patient population. **Methods:** A total of 257 patients who received an ICD for a secondary prevention indication in the University Medical Centre Groningen (UMCG) between 1 January 2012 and 31 December 2018 were retrospectively included in a consecutive manner. Appropriate ICD therapy, comprising shock and antitachycardia pacing (ATP) for ventricular fibrillation (VF) or ventricular tachycardia (VT), was the primary outcome. **Results:** The patient population included 257 patients, of whom 45 (18%) were women and 212 (82%) were men. The median of the age was 64 (interquartile range (IQR) 53–72) years. During follow-up (median duration 6.2 (IQR 4.8–7.8) years), first appropriate device therapy took place in 10 (22%) patients for women and 85 (40%) patients for men. Female sex was negatively associated with the rate of appropriate ICD therapy, univariably (hazard ratio (HR) 0.48 [95% confidence interval (CI) 0.25–0.93]; *p* = 0.030) and multivariably (HR 0.44 [95% CI 0.20–0.95]; *p* = 0.036). **Conclusions:** Women with secondary prevention ICDs were less likely than men to receive appropriate ICD therapy.

## 1. Introduction

Sudden cardiac death (SCD) is a major cause of death worldwide, responsible for up to 20% of all deaths. The majority of patients with SCD have ischemic heart disease as underlying cause [1]. Since there is a high risk of recurrence of ventricular arrhythmias, such as ventricular fibrillation (VF) and ventricular tachycardia (VT), among SCD survivors [2], current guidelines recommend the implantation of an implantable cardioverter-defibrillator (ICD) for secondary prevention in patients with documented VF or VT with hemodynamic instability without reversible causes [3]. ICDs are known to significantly reduce arrhythmic and all-cause mortality in secondary prevention [4].

Several sex differences exist in ICD populations. First, there is a sex difference with regard to the incidence of SCD, which occurs less frequently in women than it does in men. In addition, women are less likely to have VF or VT as the presenting arrhythmia and are also less frequently resuscitated by a bystander [5]. In addition to the aforementioned differences, sex differences also exist in patients treated with an ICD. Several studies have shown that women were less likely than men to receive an ICD for primary and secondary prevention [6,7,8]. This raises the concern that women may be undertreated. On the other hand, women with ICDs experience fewer appropriate ICD therapies than men, possibly indicating a less favourable risk-to-benefit ratio [9,10,11,12,13].

Most studies investigating sex differences in outcomes of patients with an ICD have been performed in primary prevention ICD recipients or a population of mixed primary and secondary prevention ICD recipients. Whether the observed sex differences also exist in a secondary prevention ICD population is less clear. Considering the fact that women with a secondary prevention ICD indication have already experienced VF or a sustained VT, it could be reasoned that they have already proved to be at risk of a recurrence of a potentially life-threatening ventricular arrhythmia, thereby necessitating the implantation of an ICD. However, there is currently a lack of studies investigating this.

Investigating sex differences in outcomes of patients with an ICD is of importance, since it might shed light on whether men and women can be expected to have similar experiences with their ICD, especially in the secondary prevention population, where this has not yet been extensively studied.

In this study, we sought to investigate sex differences in outcomes of patients with an ICD for the secondary prevention of SCD.

## 2. Materials and Methods

### 2.1. Study Population

Most of the methods described below have also been described in our previously conducted study [14].

**Figure 1:**
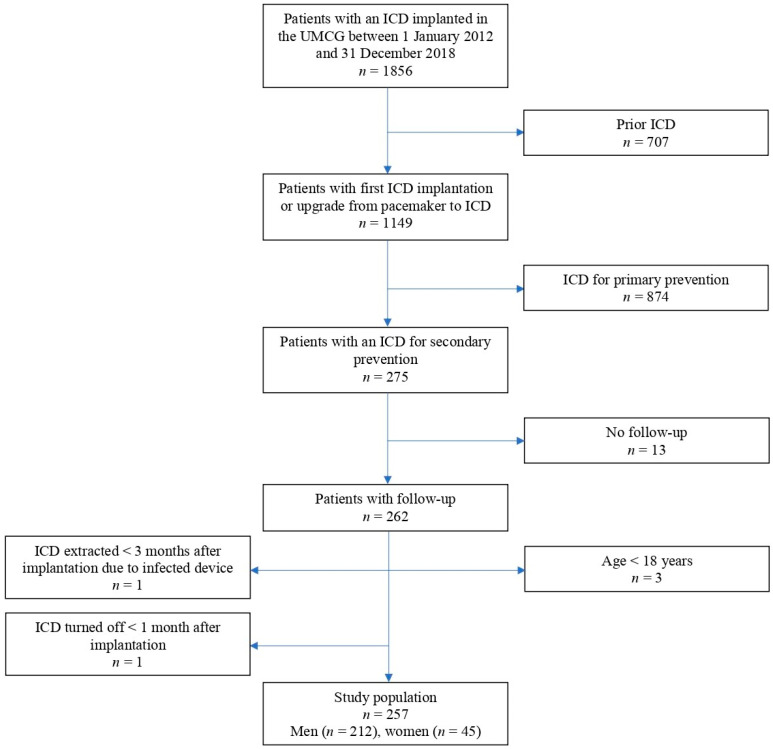
Flowchart of the patient selection procedure. ICD, implantable cardioverter-defibrillator; SCD, sudden cardiac death; UMCG, University Medical Centre Groningen.

In this observational, retrospective, single-centre study, all patients (*n* = 257) who received an ICD with the indication being secondary prevention of SCD in the University Medical Centre Groningen (UMCG) between 1 January 2012 and 31 December 2018 were consecutively included. A patient was eligible for inclusion when they fulfilled all of the following inclusion criteria: first implantation of an ICD or upgrade from pacemaker to ICD in the aforementioned period, a secondary prevention indication for the implantation of an ICD, defined as a prior documented sustained VT or VF, and age above or equal to 18 years. Exclusion criteria were ICD replacement or upgrade from one type of ICD to another, a primary prevention ICD indication, absence of follow-up data, age below 18 years, ICD extraction within a period of 3 months post-implantation because of an infected device, and the ICD being turned off within a period of 1 month post-implantation (see Figure 1). The ICD indication was based on the ESC guidelines and was set in multidisciplinary team meetings [3].

A waiver indicating that this study does not fall under the scope of the Medical Research Involving Human Subjects Act was obtained from the UMCG Medical Ethical Committee (METc 2023/141).

### 2.2. Data Acquisition and Outcomes

Data were obtained at baseline, which was defined as the moment the ICD was implanted. Data on aetiology of presenting ventricular arrhythmia, medical history, medication use, laboratory measurements, echocardiography and electrocardiography were obtained. Visits for follow-up took place every half year or every year. The primary outcome of this study was the first occurrence of appropriate ICD therapy during the period of follow-up, which was defined as the combination of shock and antitachycardia pacing (ATP) applied for VT or VF. Data on this outcome were obtained from ICD recordings. If available, home monitoring was used. The first appropriate shock, appropriate ATP and inappropriate shock, as well as all-cause mortality were selected as secondary outcomes. Inappropriate shocks were defined as shocks that were applied in the absence of VT or VF, including shocks that were given for regular supraventricular tachycardia, atrial fibrillation, noise or T wave oversensing. All therapies were assessed for appropriateness retrospectively or at the moment the therapy occurred based on electrogram recordings. Device-related complications, which included lead failure, infection and perioperative complications such as pneumothorax, lead dislocation, bleeding and cardiac perforation occurring within 90 days after implantation of the ICD, were obtained from the electronic medical records. For all events, the time until their first occurrence was obtained.

### 2.3. ICD Programming

In patients who had a monomorphic VT as the presenting ventricular arrhythmia, the ICD device was programmed to have a zone of therapy from a heart rate based on a cycle length 20 ms longer than that of the VT that was the index arrhythmia, but not faster than 200 beats per minute with prior to detection 30 intervals. ICDs were programmed to deliver 2 burst ATPs with in the second ATP burst decreasing cycle length, which was then followed by maximal output shocks. A second ICD zone of therapy was programmed from 230 beats per minute with prior to detection 30 intervals, ATP while charging, then followed by maximal output shocks. In patients who had VF as the presenting ventricular arrhythmia, ICD programming was as follows: zone of therapy from 188 or 200 beats per minute with prior to detection 30 intervals, with the ICD being programmed to deliver 2 burst ATPs with in the second ATP burst decreasing cycle length, with shocks then following at a maximal output; then a second ICD zone of therapy from 230 beats per minute with 30 intervals prior to detection, ATP while charging, then followed by maximal output shocks.

### 2.4. Statistical Analysis

Categorical and dichotomous data are presented as numbers and percentages. Mean and standard deviation are given for continuous data with a normal distribution. In case the distribution was skewed, the median is given with the interquartile range (IQR). To compare men and women with respect to the baseline characteristics, a chi-square or Fisher’s exact test, a *t*-test for independent groups and Mann–Whitney test were performed where appropriate.

Cumulative event-free survival was assessed for all outcomes using log-rank tests and Kaplan–Meier curves. Univariable Cox regression analyses were performed, after which we adjusted for potential confounders in multivariable analyses, the confounders being chosen based on theory. Potential confounders consisted of age, ventricular arrhythmia of unclear cause, index ventricular arrhythmia, body mass index, prior atrial fibrillation, prior non-sustained VT, prior syncope, prior myocardial infarction, estimated glomerular filtration rate, QRS fragmentation and ejection fraction of the left ventricle. The adjusted Cox regression analysis for the primary outcome was repeated for the two main aetiological subgroups, those being ischemic heart disease and non-ischemic heart disease, to determine whether a statistically significant difference between women and men could be detected within these subgroups. Schoenfeld residuals were used to check the proportional hazards assumption. Multicollinearity was checked using Cramer’s V and Phi coefficient for categorical and dichotomous variables, and for continuous variables, Spearman’s and Pearson’s correlation coefficients, and was considered absent in case of a coefficient <0.7. In addition, variance inflation factor (VIF) was lower than 4, with the tolerance being higher than 0.25. Finally, the presence of first-line interactions was checked for, and no significant interactions were found. We performed a sensitivity analysis using fifty multiply imputed datasets as a way of accounting for missing data.

Statistical significance was defined by a *p*-value < 0.05. We performed all the statistical analyses with SPSS version 28.0 (SPSS Institute, Chicago, IL, USA), as well as with Stata 17.

## 3. Results

### 3.1. Characteristics of Patient Population

**Figure 2:**
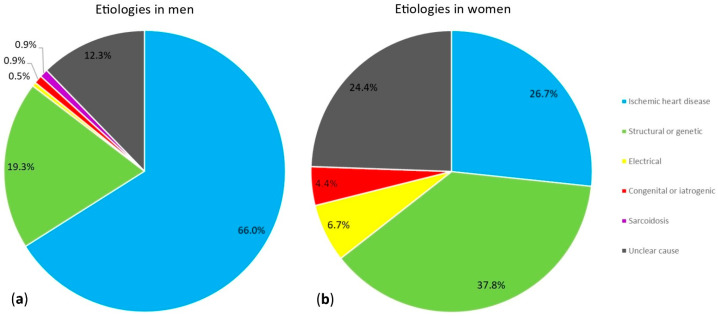
Two pie charts showing the aetiological groups of index ventricular arrhythmia in men (**a**) and women (**b**).

The study population comprised 257 patients, of whom 45 (18%) were women. The median of the age was 64 (IQR 53–72) years, which was different for women (58 years (IQR 47–67 years)) than for men (65 years (IQR 55–72 years)) (*p* = 0.003). The median left ventricular ejection fraction was 47% (35–58%) in women and 43% (34–53%) in men (*p* = 0.129). A total of 12 (27%) women and 140 (66%) men had ischemic heart disease (*p* < 0.001). Ventricular arrhythmia with an unclear cause, referring to ventricular arrhythmia of unknown cause as well as ventricular arrhythmia with a potential but uncertain underlying substrate, was significantly different between women and men (11 (24%) in women vs. 26 (12%) in men; *p* = 0.035) (Table 1). A detailed presentation of the aetiologies of the presenting ventricular arrhythmia in women and in men can be found in Figure 2. A more detailed overview of the aetiologies for the total population can be found in our previously conducted study [14]. A more comprehensive list of patient characteristics is displayed in Table S1.

### 3.2. Outcomes during Follow-Up

The median duration of the period of follow-up was 6.2 (IQR 4.8–7.8) years. This duration did not differ significantly between the two sexes (7.0 (IQR 4.9–8.3) years for women, 6.0 (IQR 4.8–7.6) years for men; *p* = 0.209). The number of patients with first appropriate therapy was 10 (22%) for women and 85 (40%) for men. The number of patients with first appropriate shock was 7 (16%) for women and 65 (31%) for men. A total of 8 (18%) women and 70 (33%) men had a first occurrence of ATP. A first occurrence of inappropriate ICD therapy occurred in 5 (11%) women and 21 (10%) men, and a first occurrence of inappropriate shock occurred in 3 (7%) women and 14 (7%) men. A total of 6 (13%) women and 53 (25%) men died. A total of 4 (9%) women and 16 (8%) men experienced device-related complications (Table 2).

### 3.3. Sex Differences in Outcomes

**Figure 3:**
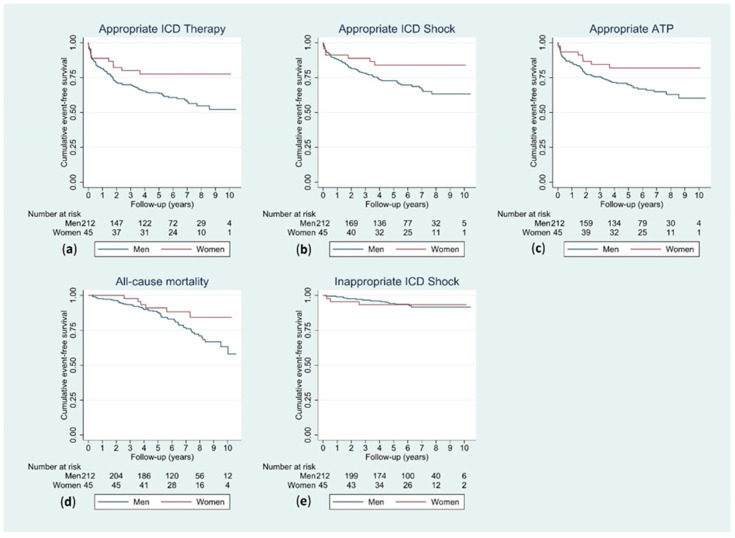
Kaplan–Meier curves for men and women for the outcomes of appropriate ICD therapy (**a**), appropriate ICD shock (**b**), appropriate ATP (**c**), all-cause mortality (**d**) and inappropriate ICD shock (**e**). ATP, antitachycardia pacing; ICD, implantable cardioverter-defibrillator.

Cumulative appropriate ICD therapy-free survival was significantly higher in women than it was in men (log-rank *p* = 0.026). The same was true for appropriate shock (log-rank *p* = 0.042) and appropriate ATP (log-rank *p* = 0.041). No significant difference was observed for the outcomes of death (log-rank *p* = 0.079) and inappropriate shock (log-rank *p* = 0.982) (Figure 3).

Female sex was negatively associated with the rate of appropriate device therapy, univariably (hazard ratio (HR) 0.48 [95% confidence interval (CI) 0.25–0.93]; *p* = 0.030) and after adjustment for potential confounders (HR 0.44 [95% CI 0.20–0.95]; *p* = 0.036). Female sex was significantly associated with appropriate ATP in multivariable analysis (HR 0.40 [95% CI 0.16–1.00]; *p* = 0.049), but not with appropriate shock (HR 0.50 [95% CI 0.21–1.20]; *p* = 0.122) (Table 3). Our sensitivity analysis with multiple imputation showed similar results, with a significant association between female sex and appropriate ICD therapy (Table S2). The observed sex difference for the primary outcome was also found within the aetiological subgroup of non-ischemic heart disease, but could not be demonstrated within the group of patients with ischemic heart disease (Table S3). No significant association was found between female sex and all-cause mortality (HR 1.12 [95% CI 0.45–2.79]; *p* = 0.805) or inappropriate ICD shock (HR 1.28 [95% CI 0.33–4.92]; *p* = 0.717) (Table 3). For an overview of the Cox regression analysis results for the potential confounders for the primary outcome, see Table S4.

## 4. Discussion

In this retrospective study, we found that women were less likely to experience appropriate ICD therapy and appropriate ATP than men. No sex differences were found for appropriate or inappropriate shock and all-cause mortality.

Female sex was associated with fewer appropriate ICD therapies. This association remained significant in our secondary analysis using multiple imputation. Women also had fewer first occurrences of appropriate ATP. Our findings are consistent with findings from previous studies, although most studied populations consisted of either primary prevention or mixed primary and secondary prevention ICD recipients [9,11,13,15,16]. Styles et al. included patients with an ICD for both primary (*n* = 511) and secondary (*n* = 265, of whom 51 were women) prevention indication and found that women had a lower likelihood of receiving appropriate ICD therapy than men in secondary prevention ICD recipients, but not in primary prevention ICD recipients [13]. The observed sex difference for the primary outcome was also found when considering only patients with non-ischemic heart disease. However, such a difference could not be demonstrated for the aetiology of ischemic heart disease. This seems to be somewhat consistent with the findings of Saxena et al., who found that women experience fewer ventricular tachyarrhythmias than men and that this difference is greater in patients with non-ischemic heart disease [10]. However, other studies do not show this [17].

No difference was found between women and men with regard to appropriate or inappropriate shock and all-cause mortality. This is in agreement with previous studies, which also did not find a sex difference for inappropriate shock [9,12,13], nor for all-cause mortality [9,10,12,13,16], although one study did show a significant difference in mortality, with a lower mortality among women [11]. Earlier studies have demonstrated a lower cumulative rate of appropriate shock in women, although these did not have a patient population with only secondary prevention ICD recipients [9,10,11,12,15].

A potential explanation for the findings of this study may be as follows. The observed difference between women and men with regard to the outcome of appropriate ICD therapy may be hormonally determined and be due to pre-menopausal cardiovascular protection in younger women, which may be lost after the occurrence of menopause. A similar idea has been proposed by Skjelbred et al. in the context of SCD, the incidence rates of which have been found to differ between women and men. The greatest difference was seen among younger and middle-aged people. The proposed explanation for this finding was the loss of cardiovascular protection offered by oestrogen in postmenopausal women [18]. Another finding supporting this hypothesis was reported by Styles et al., who found that although women were less likely to receive appropriate device therapy than men in younger patients, no such difference between the sexes existed in older patients [13]. This potential explanation for our findings remains speculative, however.

The translation of the findings of this study to the implications for patients and their care must proceed with caution. Our finding that women, compared to men, are less likely to receive appropriate ICD therapy does not mean that women do not benefit from an ICD. As previously mentioned, 22% of women received appropriate therapy during a follow-up period of 6.2 years, which is still a considerable number. In addition, it is important to note that the observed sex difference in the rate of appropriate ICD therapies does not translate into a difference in survival benefit. Studies that did investigate sex differences in survival benefit, which were mostly conducted with primary prevention ICD recipients, yielded somewhat conflicting results [15,16,19], but overall, women seem to benefit less from an ICD than men [20]. In SCD-HeFT, which included a population of primary prevention ICD recipients of whom 23% were female, the absolute reduction in mortality at five years was approximately 7 percent, while the rate of ICD therapies delivered for fast VT or VF was 21% [21]. In a subsequent substudy, it was found that the ICD benefit was less for women than for men [15,22]. In our study, after adjusting for potential confounders, the outcome of appropriate ATP was significantly different between women and men, but this was not the case for the outcome of appropriate shock. Although ATP is also given for and can terminate fast VTs, they are generally given for slower VTs, with shocks conventionally given for faster VTs or VF [23]. Since the latter may be of greater importance in the discussion concerning benefit derived from an ICD, no conclusions can be drawn with regard to benefit derived from the ICD based on our findings.

Although the implications for patients based on this study are still very limited, this study does generate the hypothesis that the outcomes, more specifically the outcomes of appropriate device therapy and appropriate ATP, of women with an ICD implanted for secondary prevention indications differ from those of men. Further research is necessary prior to any discussion regarding implications for patients, especially since no conclusions can be drawn with regard to benefit derived from the ICD. To further investigate the benefit of ICDs for secondary prevention among women, large prospective studies are needed to compare women with a prior VF or sustained VT with and without an ICD with regard to outcomes such as arrhythmic deaths and all-cause mortality. Future studies should also be conducted to investigate whether our finding that women were less likely to receive appropriate device therapy than men is also observed in a prospective setting, as well as whether such a sex difference exists irrespective of aetiology of presenting arrhythmia. To shed further light on the influence of differences in baseline characteristics between the sexes, including age and underlying aetiology, on our findings, prospective studies with similar baseline characteristics for women and men are required. Finally, further research is warranted to find predictors of ICD benefit so as to determine which patients in the female population are most likely to benefit from the implantation of an ICD and which patients are least likely.

This study contributes to the limited data on sex differences in secondary prevention ICD recipients. Some previously conducted studies that did find sex differences did not specifically focus on this as their primary research question. Furthermore, most studies investigating sex differences in outcomes of patients with an ICD that have been conducted in the past have not specifically focused on a secondary prevention population, including either a mixed primary and secondary prevention ICD population or focusing entirely on patients with an ICD for primary prevention. For instance, only about 28% of the total study population of 6021 patients included in the study conducted by MacFadden et al. had a secondary prevention ICD indication, of whom approximately 22% were women [9]. Wijers et al. investigated a study population of 1075 patients with 26% women and 39% having a secondary prevention ICD indication [11]. Wilson et al. included 137 patients of whom 74% had an ICD for secondary prevention, of whom 25% were women [12]. The study conducted by Styles et al. included 776 patients with 34% secondary prevention patients, of whom 19% were women [13].

Despite the fact that this study contributes to the scant existing data on sex differences in a secondary prevention ICD population, several limitations are worth mentioning. The number of women in this study was low (*n* = 45). Even though this might be a reflection of the real-world situation, this is still an important limitation. Our moderate sample size precluded robust conclusions and our findings are therefore only hypothesis-generating. Bias could have resulted from missing data. Multiple imputation was used as a method to deal with missing data, thereby minimising bias as a result of missing data. Differences in baseline characteristics between women and men, such as the significantly different age as well as the different underlying aetiologies between the two sexes, might at least partially be responsible for the observed sex difference in the outcomes, which is why future studies are required to determine whether the observed sex difference is an actual sex difference and not a result of the difference in risk factors between women and men. To account for this difference in this study, potential confounders were adjusted for in multivariable analyses, reducing the risk of bias. However, residual confounding may still be present.

## 5. Conclusions

Women were significantly less likely than men to receive appropriate ICD therapy, although a considerable number of women still received appropriate therapy.

## Figures and Tables

**Table 1:** Baseline characteristics.

Variable	Men (*n* = 212)	Women (*n* = 45)	*p*-Value
Age (years)	64.9 (54.8–72.1)	57.7 (47.3–66.9)	**0.003**
Ischemic heart disease	140 (66.0%)	12 (26.7%)	**<0.001**
Non-ischemic heart disease	46 (21.7%)	22 (48.9%)	**<0.001**
Ventricular arrhythmia of unclear cause	26 (12.3%)	11 (24.4%)	**0.035**
Presenting ventricular arrhythmia			0.651
VF	144 (67.9%)	29 (64.4%)	
Sustained VT	68 (32.1%)	16 (35.6%)	
BMI (kg/m^2^)	26.5 (24.5–29.2)	27.0 (23.9–34.8)	0.423
NYHA class			1.000
I or II	147 (69.3%)	28 (62.2%)	
III or IV	25 (11.8%)	5 (11.1%)	
Medical history			
Myocardial infarction	126 (59.4%)	11 (24.4%)	**<0.001**
DM	40 (18.9%)	8 (17.8%)	0.865
Syncope	18 (8.5%)	6 (13.3%)	0.394
Prior heart surgery	65 (30.7%)	1 (2.2%)	**<0.001**
Atrial fibrillation	74 (34.9%)	12 (26.7%)	0.287
Non-sustained VT	40 (18.9%)	7 (15.6%)	0.602
CAD	145 (68.4%)	12 (26.7%)	**<0.001**
Echocardiography			
LVEF (%)	43 (34–53)	47 (35–58)	0.129
Electrocardiography			
Rhythm			**0.024**
Sinus rhythm	179 (84.4%)	42 (93.3%)	
Atrial fibrillation	27 (12.7%)	1 (2.2%)	
Atrial flutter	2 (0.9%)	0 (0.0%)	
Pacemaker	1 (0.5%)	2 (4.4%)	
QRS fragmentation †	79 (37.3%)	11 (24.4%)	0.214
Laboratory values			
eGFR (mL/min/1.73 m^2^)	76 ± 23	83 ± 24	0.101
Medication at baseline			
ACE-I/ARB	99 (46.7%)	14 (31.1%)	0.117
β-blocker	106 (50.0%)	19 (42.2%)	0.606
Calcium antagonist	39 (18.4%)	4 (8.9%)	0.166
Diuretic	50 (23.6%)	6 (13.3%)	0.191
Statin	100 (47.2%)	11 (24.4%)	**0.012**
MRA	19 (9.0%)	3 (6.7%)	1.000
Class 3 antiarrhythmic drugs	13 (6.1%)	2 (4.4%)	1.000
Digoxin	8 (3.8%)	1 (2.2%)	1.000

Data are given as *n* (%) in case the data are categorical, median with interquartile range (IQR) when the data are continuous and the distribution is skewed, and mean with standard deviation (SD) when the data are continuous and normally distributed. The presented numbers sometimes do not add up to a total of 100% because of missing data. The given *p*-values are a reflection of the difference between women and men. *p*-values < 0.05 are presented in bold. ACE-I, angiotensin-converting enzyme inhibitor; ARB, angiotensin receptor blocker; BMI, body mass index; CAD, coronary artery disease; DM, diabetes mellitus; eGFR, estimated glomerular filtration rate; LVEF, left ventricular ejection fraction; MRA, mineralocorticoid receptor antagonist; NYHA, New York Heart Association; VF, ventricular fibrillation; VT, ventricular tachycardia. † QRS complex could be fragmented, unfragmented or broad (>120 ms).

**Table 2:** Number of patients with outcomes during follow-up.

Outcomes	Men (*n* = 212)	Women (*n* = 45)
Appropriate ICD therapy	85 (40.1%)	10 (22.2%)
Appropriate shock	65 (30.7%)	7 (15.6%)
Appropriate ATP	70 (33.0%)	8 (17.8%)
All-cause mortality	53 (25.0%)	6 (13.3%)
Cardiac cause of death	13 (6.1%)	1 (2.2%)
Non-cardiac cause of death	11 (5.2%)	3 (6.7%)
Unknown cause of death	29 (13.7%)	2 (4.4%)
Inappropriate ICD therapy	21 (9.9%)	5 (11.1%)
Inappropriate shocks	14 (6.6%)	3 (6.7%)
Complications	16 (7.5%)	4 (8.9%)
Lead failure	11 (5.2%)	1 (2.2%)
Perioperative complications	6 (2.8%)	2 (4.4%)
Infection	0 (0.0%)	0 (0.0%)
Other complications	0 (0.0%)	1 (2.2%)

Data represent the first incident of the outcomes and are expressed as *n* (%). ATP, antitachycardia pacing; ICD, implantable cardioverter-defibrillator.

**Table 3:** Univariable and adjusted Cox regression analyses with female sex as determinant.

	Univariable		Adjusted	
Outcomes	HR (95% CI)	*p*-Value	HR (95% CI)	*p*-Value
Appropriate therapy	0.48 (0.25–0.93)	0.030	0.44 (0.20–0.95) †	0.036
Appropriate shock	0.45 (0.21–0.99)	0.048	0.50 (0.21–1.20) †	0.122
Appropriate ATP	0.48 (0.23–0.99)	0.046	0.40 (0.16–1.00) †	0.049
All-cause mortality	0.48 (0.20–1.11)	0.086	1.12 (0.45–2.79) ‡	0.805
Inappropriate shock	0.99 (0.28–3.43)	0.982	1.28 (0.33–4.92) §	0.717

CI, confidence interval; HR, hazard ratio. † Adjusted for age, unclear cause of ventricular arrhythmia, index ventricular arrhythmia, BMI, prior atrial fibrillation, prior non-sustained VT, prior syncope, prior myocardial infarction, eGFR, QRS fragmentation and left ventricular ejection fraction. ‡ Adjusted for age, unclear cause of ventricular arrhythmia, diabetes, index ventricular arrhythmia, prior heart surgery, prior myocardial infarction and eGFR. § Adjusted for age, unclear cause of ventricular arrhythmia and rhythm on ECG.

## Data Availability

The data which are presented in this study are available upon request from the corresponding author.

## Supplementary Materials

The following supporting information can be downloaded at: https://www.mdpi.com/article/10.3390/jcdd11040116/s1, Table S1: Baseline characteristics; Table S2: Adjusted Cox regression analyses with female sex as determinant using multiple imputation; Table S3: Adjusted Cox regression analyses for the primary outcome with female sex as determinant separately for ischemic and non-ischemic heart disease; Table S4: Adjusted Cox regression analyses for the primary outcome with female sex as determinant, also showing the analysis results for the potential confounders.
