# Supplementary material for: Sex Differences in Outcomes of Patients with an Implantable Cardioverter-Defibrillator for the Secondary Prevention of Sudden Cardiac Death
Source: J Cardiovasc Dev Dis. 2024 Apr 5;11(4):116. doi: 10.3390/jcdd11040116 (PMC11050510; doi:10.3390/jcdd11040116)
Supplement: Supplementary file 1 [file jcdd-11-00116-s001.zip › jcdd-2914046-supplementary.pdf]

**Table S1. Baseline characteristics.**			
**Variable**	**Men (*n* = 212)**	**Women (*n* = 45)**	***P*-value**
Age (years)	64.9 (54.8 – 72.1)	57.7 (47.3 – 66.9)	**0.003**
Female sex			
Ischemic heart disease	140 (66.0%)	12 (26.7%)	**<0.001**
Non-ischemic heart disease	46 (21.7%)	22 (48.9%)	**<0.001**
Ventricular arrhythmia of unclear cause	26 (12.3%)	11 (24.4%)	**0.035**
Presenting ventricular arrhythmia			0.651
VF	144 (67.9%)	29 (64.4%)	
Sustained VT	68 (32.1%)	16 (35.6%)	
OHCA	151 (71.2%)	31 (68.9%)	0.754
Type of ICD			**0.031**
S-ICD	7 (3.3%)	5 (11.1%)	
VVI-ICD	107 (50.5%)	14 (31.1%)	
DDD-ICD	70 (33.0%)	18 (40.0%)	
CRT-D	28 (13.2%)	8 (17.8%)	
BMI (kg/m^2)	26.5 (24.5 – 29.2)	27.0 (23.9 – 34.8)	0.423
NYHA class			1.000
I or II	147 (69.3%)	28 (62.2%)	
III or IV	25 (11.8%)	5 (11.1%)	
Medical history			
Hypertension	106 (50.0%)	18 (40.0%)	0.223
Dyslipidemia	89 (42.0%)	7 (15.6%)	**<0.001**
Myocardial infarction	126 (59.4%)	11 (24.4%)	**<0.001**
DM	40 (18.9%)	8 (17.8%)	0.865
Family history SCD	19 (9.0%)	5 (11.1%)	0.583
Syncope	18 (8.5%)	6 (13.3%)	0.394
Prior heart surgery	65 (30.7%)	1 (2.2%)	**<0.001**
Atrial fibrillation	74 (34.9%)	12 (26.7%)	0.287
Non-sustained VT	40 (18.9%)	7 (15.6%)	0.602
CAD	145 (68.4%)	12 (26.7%)	**<0.001**
Echocardiography			
LVEF (%)	43 (34 – 53)	47 (35 – 58)	0.129
LAVI (mL/m^2)	37 (29 – 45)	34 (29 – 39)	0.223
Maximum myocardial wall thickness (mm)	11.1 ± 2.1	10.0 ± 1.7	**0.003**
LVEDD (mm)	55.5 ± 8.4	53.1 ± 8.6	0.115
LV mass (g)	222 ± 64	172 ± 48	**<0.001**
RVF			0.878
Poor	3 (1.4%)	0 (0.0%)	
Mediocre	6 (2.8%)	0 (0.0%)	
Reasonable	30 (14.2%)	6 (13.3%)	
Good	160 (75.5%)	35 (77.8%)	
Mitral insufficiency			0.890
None	125 (59.0%)	28 (62.2%)	
Mild (grade 1)	49 (23.1%)	12 (26.7%)	
Moderate (grade 2)	17 (8.0%)	3 (6.7%)	
Severe (grade 3)	5 (2.4%)	0 (0.0%)	
LGE on CMR			**0.013**
Yes	72 (34.0%)	8 (17.8%)	
No	39 (18.4%)	14 (31.1%)	
Electrocardiography			
Rhythm			**0.024**
Sinus rhythm	179 (84.4%)	42 (93.3%)	
Atrial fibrillation	27 (12.7%)	1 (2.2%)	
Atrial flutter	2 (0.9%)	0 (0.0%)	

Pacemaker	1 (0.5%)	2 (4.4%)	
Ventricular heart rate (bpm)	67 ± 14	71 ± 15	0.064
PR interval (ms)	169 (152 – 189)	165 (151 – 186)	0.450
QRS duration (ms)	109 (98 – 128)	106 (90 – 130)	0.349
QRS axis (degrees)	30 (-15 – 65)	40 (-4 – 68)	0.337
QT interval (ms)	435 ± 52	431 ± 46	0.648
QRS fragmentation†	79 (37.3%)	11 (24.4%)	0.214
QRS morphology			0.292
Normal	113 (53.3%)	27 (60.0%)	
LBBB	17 (8.0%)	8 (17.8%)	
LAFB	35 (16.5%)	3 (6.7%)	
LPFB	3 (1.4%)	1 (2.2%)	
RBBB	13 (6.1%)	1 (2.2%)	
RBBB + LAFB	9 (4.2%)	1 (2.2%)	
RBBB + LPFB	3 (1.4%)	0 (0.0%)	
IVCD	15 (7.1%)	3 (6.7%)	
Pacing	2 (0.9%)	1 (2.2%)	
Early repolarization	13 (6.1%)	2 (4.4%)	1.000
Laboratory values			
Hb (mmol/L)	8.2 ± 1.2	7.9 ± 0.9	0.122
Na^+ (mmol/L)	140 (138 – 141)	140 (139 – 142)	0.586
K^+ (mmol/L)	4.2 ± 0.4	4.2 ± 0.4	0.438
Creatinine (µmol/L)	89 (78 – 104)	72 (58 – 84)	**<0.001**
eGFR (mL/min/1.73m^2)	76 ± 23	83 ± 24	0.101
Urea (mmol/L)	6.3 (5.0 – 8.1)	5.4 (4.3 – 6.8)	**0.002**
NT-proBNP (ng/L)	737 (247 – 1,753)	404 (166 – 1,058)	0.062
Medication at baseline			
ACE-I/ARB	99 (46.7%)	14 (31.1%)	0.117
β-blocker	106 (50.0%)	19 (42.2%)	0.606
Calcium antagonist	39 (18.4%)	4 (8.9%)	0.166
Diuretic	50 (23.6%)	6 (13.3%)	0.191
Statin	100 (47.2%)	11 (24.4%)	**0.012**
MRA	19 (9.0%)	3 (6.7%)	1.000
Class 1 antiarrhythmic drugs	0 (0.0%)	1 (2.2%)	0.164
Class 3 antiarrhythmic drugs	13 (6.1%)	2 (4.4%)	1.000
Digoxin	8 (3.8%)	1 (2.2%)	1.000

Data are given as *n* (%) in case the data is categorical, median with interquartile range (IQR) when the data is continuous and the distribution is skewed, and mean with standard deviation (SD) when the data is continuous and normally distributed. The presented numbers sometimes do not add up to a total of 100% because of missing data. The given *P*-values are a reflection of the difference between women and men. *P*-values <0.05 are presented in bold. ACE-I, angiotensin-converting enzyme inhibitor, ARB, angiotensin receptor blocker, BMI, body mass index, CAD, coronary artery disease, CMR, cardiac magnetic resonance, CRT-D, cardiac resynchronization therapy ICD, DDD-ICD, dual-chamber ICD, DM, diabetes mellitus, eGFR, estimated glomerular filtration rate, Hb, hemoglobin, ICD, implantable cardioverter-defibrillator, IVCD, intraventricular conduction delay, LAFB, left anterior fascicular block, LAVI, left atrial volume index, LBBB, left bundle branch block, LGE, late gadolinium enhancement, LPFB, left posterior fascicular block, LV, left ventricle, LVEDD, left ventricular end-diastolic diameter, LVEF, left ventricular ejection fraction, MRA, mineralocorticoid receptor antagonist, NT-proBNP, N-terminal pro-brain natriuretic peptide, NYHA, New York Heart Association, OHCA, out-of-hospital cardiac arrest, RBBB, right bundle branch block, RVF, right ventricular function, SCD, sudden cardiac death, S-ICD, subcutaneous ICD, VF, ventricular fibrillation, VT, ventricular tachycardia, VVI-ICD, single-chamber ICD.

† QRS complex could be fragmented, unfragmented or broad (>120ms).

**Table S2. Adjusted Cox regression analyses with female sex as determinant using multiple imputation.**	
	**Adjusted**

Outcomes	HR (95% CI)	*P*-value
Appropriate therapy	0.49 (0.24 – 0.99)†	0.047
Appropriate shock	0.52 (0.23 – 1.18)†	0.117
Appropriate ATP	0.48 (0.21 – 1.08)†	0.075
All-cause mortality	1.01 (0.40 – 2.50)‡	0.991
Inappropriate shock	1.28 (0.33 – 4.92)§	0.717

CI, confidence interval, HR, hazard ratio.
† Adjusted for age, unclear cause of ventricular arrhythmia, index ventricular arrhythmia, BMI, prior atrial fibrillation, prior non-sustained VT, prior syncope, prior myocardial infarction, eGFR, QRS fragmentation and left ventricular ejection fraction.
‡ Adjusted for age, unclear cause of ventricular arrhythmia, diabetes, index ventricular arrhythmia, prior heart surgery, prior myocardial infarction, and eGFR.
§ Adjusted for age, unclear cause of ventricular arrhythmia and rhythm on ECG.

Table S3. Adjusted Cox regression analyses for the primary outcome with female sex as determinant separately for ischemic and non-ischemic heart disease.

	HR (95% CI)	*P*-value
Female sex (IHD)	0.55 (0.19 – 1.55)	0.257
Female sex (NIHD)	0.08 (0.01 – 0.49)	0.006

The analyses were adjusted for the same potential confounders as indicated before for the primary outcome. CI, confidence interval, HR, hazard ratio, IHD, ischemic heart disease, NIHD, non-ischemic heart disease.

Table S4. Adjusted Cox regression analyses for the primary outcome with female sex as determinant, also showing the analysis results for the potential confounders.

Variables	HR (95% CI)	*P*-value
Female sex	0.44 (0.20 – 0.95)	0.036
Age	1.00 (0.98 – 1.02)	0.740
Unclear etiology	0.37 (0.14 – 0.99)	0.048
VT as index arrhythmia	1.70 (1.05 – 2.74)	0.031
BMI	1.03 (0.99 – 1.09)	0.163
Prior atrial fibrillation	0.75 (0.46 – 1.24)	0.263
Prior non-sustained VT	1.70 (1.02 – 2.81)	0.040
Prior syncope	1.30 (0.65 – 2.61)	0.462
Prior myocardial infarction	1.10 (0.66 – 1.84)	0.701
eGFR	0.99 (0.98 – 1.01)	0.346
QRS complex		0.095
Fragmented	1.84 (1.06 – 3.19)	0.031
QRS >120ms	1.46 (0.77 – 2.76)	0.243
Left ventricular ejection fraction	1.01 (0.99 – 1.03)	0.396

BMI, body mass index, CI, confidence interval, eGFR, estimated glomerular filtration rate, HR, hazard ratio, VT, ventricular tachycardia.
